# Association between splenic volume changes and prognosis in advanced gastric cancer patients receiving chemotherapy combined with immunotherapy

**DOI:** 10.3389/fonc.2026.1763993

**Published:** 2026-02-11

**Authors:** Zhiying Li, Weiwei Liu, Guilin Zhang, Xiaona Fu, Yi Li, Jie Lou, Bingxin Gong, Lingli Li, Lian Yang, Peng Zhang

**Affiliations:** 1 Department of Gastrointestinal Surgery, Union Hospital, Tongji Medical College, Huazhong University of Science and Technology, Wuhan, China; 2 Department of Radiology, Union Hospital, Tongji Medical College, Huazhong University of Science and Technology, Wuhan, China; 3 Hubei Provincial Clinical Research Center for Precision Radiology & Interventional Medicine, Wuhan, China; 4 Hubei Key Laboratory of Molecular Imaging, Wuhan, China

**Keywords:** computed tomography, gastric cancer, immune checkpoint inhibitors, prognosis, splenic volume

## Abstract

**Background and aim:**

The prognostic value of splenic volume (SV) in patients with advanced gastric cancer receiving chemotherapy combined with immunotherapy remains unclear. This study aimed to evaluate the association between CT-assessed changes in SV and clinical outcomes, exploring its potential as a prognostic biomarker.

**Methods:**

This retrospective cohort study included 138 advanced gastric cancer patients receiving chemotherapy combined with immunotherapy. Patients were stratified into an increased group (ΔSV > 14%, n = 87) and a non-increased group (ΔSV ≤ 14%, n = 51) based on the optimal cutoff value of 14% for the annualized change in splenic volume (ΔSV). Kaplan-Meier survival analysis, Cox proportional hazards models, and restricted cubic spline analyses were used to assess prognostic associations. A nomogram incorporating ΔSV was constructed for survival prediction.

**Results:**

The increased group showed significantly shorter median progression-free survival (PFS) (14.8 vs. 26.6 months, *P* < 0.001) and overall survival (OS) (18.9 vs. 30.9 months, *P* < 0.001) compared to the non-increased group. Multivariate analysis identified ΔSV >14% as an independent risk factor for both PFS (HR, 0.424 [95% CI, 0.238 - 0.755]; *P<* 0.001) and OS (HR, 0.233 [95% CI, 0.109 - 0.501]; *P* < 0.001). A nomogram integrating ΔSV, stages, and other indicators demonstrated significantly better predictive performance than a stages-only model (1-year OS AUC: 0.802 vs. 0.564; 2-year OS AUC: 0.883 vs. 0.686).

**Conclusions:**

Increased SV is associated with poorer clinical outcomes in advanced gastric cancer patients receiving chemotherapy combined with immunotherapy.

## Introduction

Gastric cancer (GC) represents a significant global health challenge, ranking as the fifth most common cancer and the fourth leading cause of cancer-related mortality worldwide (1). In recent years, immunotherapy for advanced gastric cancer has made significant progress, driven by a deeper understanding of the cancer microenvironment (2, 3). Immune checkpoint inhibitors (ICIs) have shown promising therapeutic effects in certain GC patients, particularly those with high PD-L1 expression, high microsatellite instability, and Epstein-Barr virus positivity, all of which are associated with a higher response rate to immunotherapy (4, 5). However, a significant proportion of patients do not benefit from immunotherapy and may experience associated adverse effects (6–8). Therefore, further research on immune-related biomarkers in GC is essential for the accurate selection of candidates for immunotherapy.

The spleen, as the largest secondary lymphoid organ in the human body, plays a complex and pivotal dual role in immune regulation (9). On one hand, it serves as a critical site for lymphocyte activation, antigen presentation, and the initiation of immune responses, thereby exhibiting potential anti-tumor immune-activating functions (10, 11). On the other hand, under certain pathological conditions—such as chronic inflammation or tumor progression—the spleen can give rise to a substantial population of immunosuppressive cells, including myeloid-derived suppressor cells (MDSCs) and regulatory T cells (Tregs), as well as secrete pro-inflammatory cytokines, ultimately fostering an immunosuppressive microenvironment that facilitates tumor immune escape and progression (12, 13).

Preliminary studies in other solid tumors, including metastatic renal cell carcinoma and pancreatic adenocarcinoma, have begun to uncover a potential link between increased splenic volume and shortened survival outcomes (14, 15). These findings provide initial support for the prognostic utility of ΔSV, suggesting that it may serve as a broadly applicable indicator for assessing systemic immune status and predicting the efficacy of immunotherapies. However, within the context of advanced gastric cancer—an area of high clinical importance—the specific trajectory of ΔSV during immunotherapy and its exact relationship with clinical outcomes remain unclear. In light of this gap, the present study undertakes a retrospective analysis of data from advanced gastric cancer patients receiving ICIs to determine the association between ΔSV and progression-free survival (PFS) and overall survival (OS) and to develop an individualized prognostic model integrating ΔSV with key clinical features.

## Materials and methods

The study was conducted by the guidelines outlined in the 2013 revision of the Declaration of Helsinki and received approval from the Ethics Committee of Union Hospital, Tongji Medical College, Huazhong University of Science and Technology (Institutional Review Board No. 0445). Given that the study was retrospective, the requirement to obtain written informed consent was waived.

### Study participants and design

This retrospective cohort study was conducted at Union Hospital, Tongji Medical College, Huazhong University of Science and Technology, from March 2019 to June 2024. A total of 138 gastric cancer patients undergoing immunotherapy were enrolled. Inclusion criteria were: (I) histologically or cytologically confirmed gastric cancer; (II) age over 18 years; (III) adenocarcinoma pathology type; (IV) initial diagnosis and completion of treatment at this institution; (V) abdominal CT examination performed before initiation of ICIs therapy. Exclusion criteria included: (I) presence of other malignancies; (II) history of prior surgical intervention or immunotherapy; (III) coexisting chronic liver disease, systemic infectious diseases, or other conditions that may cause splenomegaly; (IV) missing medical records or relevant data; (V) poor-quality abdominal CT scans; (VI) splenic involvement.

### Study variables and data collection

Patient demographic and clinical baseline data were retrospectively collected, covering the entire period from admission to discharge or death. Follow-up assessments were conducted every six months via telephone, with a primary focus on disease progression and mortality, and continued until the patient’s death.

CT images were obtained using Siemens or Philips medical scanners, with tube voltage set at 120 kVp and automatic tube current modulation to optimize image quality. Advanced reconstruction techniques were employed using a 512×512 matrix and 64 mm×0.625 mm collimation. The standardized reconstruction slice thickness was 1.5 mm, and slice spacing ensured consistency and measurement accuracy. Images were stored and CT values measured using the Picture Archiving and Communication System (PACS).

### Measurement of splenic volume

Baseline SV was calculated using CT scans obtained within 4 weeks before starting ICI therapy. Changes in volume were assessed during follow-up imaging after ICIs treatment. Measurements were taken on both axial and coronal reformatted CT images, with SV calculated by a radiologist (L.L.L.) with 13 years of experience, blinded to clinical data. Inter-observer reproducibility was assessed in a randomly selected subset of 30 patients. Splenic volume measurements were independently performed by a second radiologist with equivalent experience, who was blinded to the initial measurements and all clinical outcomes, using the same standardized protocol. Inter-observer agreement was evaluated using a two-way random-effects intraclass correlation coefficient with absolute agreement [ICC(A,1)].

**Figure 1:**
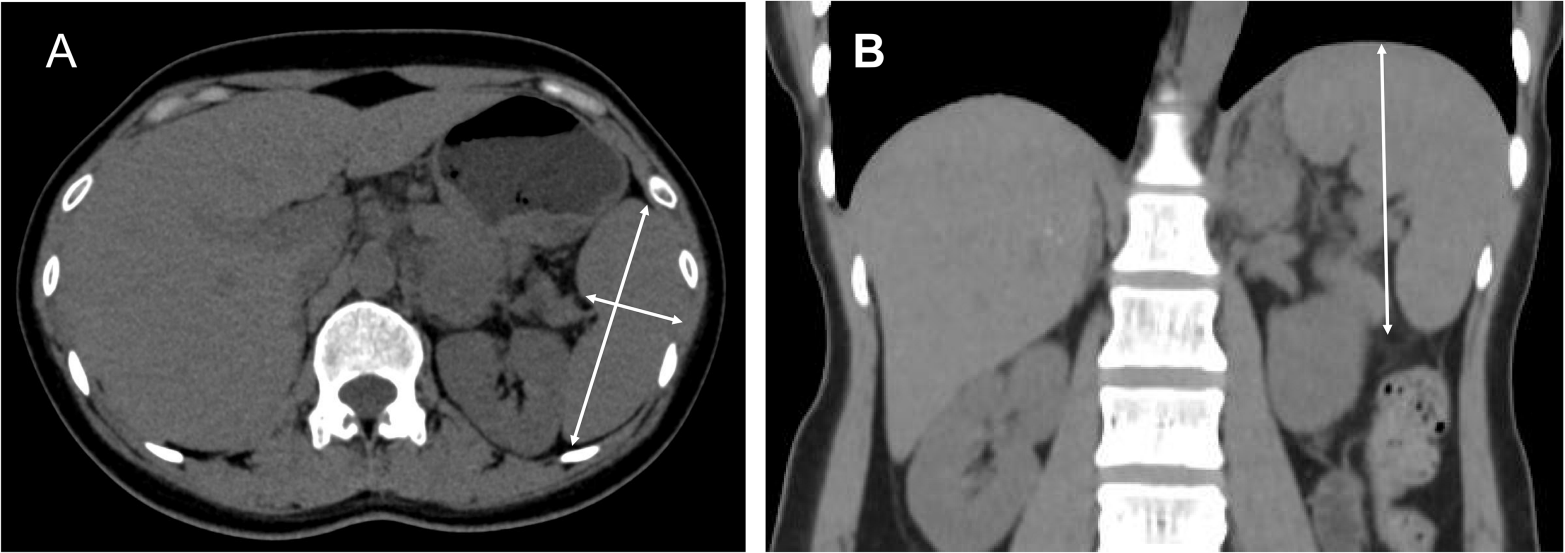
CT-based splenic volume measurement methodology. **(A)** Maximum splenic width (red line) and hilum thickness (blue line), measured perpendicular to the splenic width plane passing through the hilum. **(B)** Splenic height (yellow line), defined as the longest craniocaudal dimension.

SV was determined using the formula derived by Prassopoulos et al. The formula is as follows: $V\;\left(cm^{3}\right)\;=\;30\;+\;0.58\;\times \left(W\;\times L\;\times T\right)$. Where *W* is the maximum width at the splenic hilum, *T* is the maximum thickness of the splenic hilum in a plane perpendicular to W, and *L* is the maximum caudo-cranial length (16) (**Figure 1**).

For each patient, the volume difference of the spleen was determined by comparing the two abdominal CT images with the largest time interval. The volumetric difference was divided by the time interval in months and then multiplied by twelve to calculate the annualized volumetric evolution, expressed as follows: ΔSV (%/year) = [(b − a) / a] × (12/c) Among them, *a* is the pre-treatment splenic volume (cm³), *b* is the splenic volume at the most recent follow-up (cm³), *c* is the interval between the two measurements (months), and *ΔSV* is the annualized splenic volume change (%). The ΔSV was subsequently analyzed as a binary variable, with the optimal cutoff point (14%) serving as the critical threshold (**Supplementary Figure 1**). To evaluate potential overfitting associated with the internally derived ΔSV cutoff, internal validation of the multivariable Cox model was performed using bootstrap resampling (1,000 repetitions) and repeated 5-fold cross-validation (50 repetitions). Model discrimination was assessed using Harrell’s concordance index (C-index).

To assess the robustness of ΔSV estimation under heterogeneous follow-up intervals, sensitivity analyses were conducted. These included stratification by CT follow-up duration, derivation of duration-adjusted ΔSV using linear mixed-effects modeling, and temporal standardization by interpolating splenic volume at a uniform 6-month post-treatment time point.

### Follow-up and endpoints

Radiological response was assessed using abdominal CT scans and evaluated according to the RECIST version 1.1 criteria. OS was defined as the time from study enrollment to death. PFS was defined as the time from first treatment administration to radiographic disease progression or death, whichever occurred first. Objective response rate (ORR) included patients achieving complete response (CR) or partial response (PR), while disease control rate (DCR) included patients achieving CR, PR, or stable disease (SD).

### Statistical analysis

Statistical analyses were performed using IBM SPSS Statistics (version 28) and R software (version 4.4.2). Descriptive statistics were used to assess variable distributions. Continuous variables were expressed as mean ± standard deviation or median (interquartile range [IQR]), while categorical variables were presented as counts and percentages. The optimal cutoff values for continuous variables were determined using the survminer package in R, and these thresholds were then used to dichotomize the variables. Optimal cutoff values for neutrophil-to-lymphocyte ratio (NLR), platelet-to-lymphocyte ratio (PLR), systemic immune-inflammation index (SII), lymphocyte-to-monocyte ratio (LMR), prognostic nutritional index (PNI), ΔSV, and baseline splenic volume are summarized in **Supplementary Table 1**.

Based on the selected splenic volume threshold, patients were divided into two groups: “increased group” and “non-increased group.” Kaplan-Meier survival analysis was used to assess PFS and OS, and the log-rank test was applied to compare the two groups. Univariate and multivariate Cox regression models were used to examine the effect of ΔSV on survival. Variables with p-values less than 0.10 were included in the multivariate analysis model. Hazard ratios (HR) and corresponding 95% confidence intervals (CIs) were calculated.

## Results

### Inter-observer agreement of splenic volume measurements

Bland–Altman analysis showed that most measurements were within the 95% limits of agreement, without evident systematic bias (**Supplementary Figure 2**). Intraclass correlation coefficient analysis using a two-way random-effects model with absolute agreement yielded an ICC(A,1) of 0.938 (95% CI: 0.889–0.967; P< 0.001).

### Patient characteristics

A total of 138 gastric cancer patients were enrolled in this study (87 in the splenic volume increased group and 51 in the non-increased group), and all patients received chemotherapy combined with immune checkpoint inhibitor treatment. **Table 1** presents the baseline demographic and clinical characteristics. Among all enrolled patients, 68 (49.3%) were over 60 years old, and 70 (50.7%) were under 60. Additionally, 95 patients (68.8%) were male, and 43 (31.2%) were female. Overall, no significant differences were observed between the two groups in terms of gender, age, smoking history, disease stages, inflammatory markers, or basic biochemical indicators. Furthermore, when patients were categorized based on the median baseline splenic volume, the baseline characteristics were summarized in **Supplementary Table 2**. In the baseline volume cohort, no statistically significant association was found between splenic volume size and progression-free survival (HR, 0.74 [95% CI, 0.45–1.20]; P = 0.220) or overall survival (HR, 1.13 [95% CI, 0.65–1.98]; P = 0.664) (**Supplementary Figure 3**). Given the lack of prognostic significance of baseline splenic volume, we next focused on the prognostic value of ΔSV.

**Table 1:** Baseline characteristics of the study population stratified according to whether the splenic volume increased or not. (n=138).

Characteristics	Increased group	Non-increased group	P value
n	87	51	
Gender, n (%)			0.967
Male	60 (69%)	35 (68.6%)	
Female	27 (31%)	16 (31.4%)	
Age, n (%)			0.690
>60	44 (50.6%)	24 (47.1%)	
≤60	43 (49.4%)	27 (52.9%)	
Body mass index, n (%)			0.098
≤25	76 (87.4%)	39 (76.5%)	
>25	11 (12.6%)	12 (23.5%)	
Smoking, n (%)			0.586
No	72 (82.8%)	44 (86.3%)	
Yes	15 (17.2%)	7 (13.7%)	
Hypertension, n (%)			0.642
No	67 (77%)	41 (80.4%)	
Yes	20 (23%)	10 (19.6%)	
Cr, mean ± sd	69.387 ± 17.029	82.633 ± 88.399	0.177
Blood urea nitrogen, mean ± sd	5.1514 ± 1.6839	5.4253 ± 2.8633	0.480
Alkaline phosphatase, mean ± sd	114.72 ± 147.91	139.18 ± 159.74	0.364
Ca, mean ± sd	2.1841 ± 0.14816	2.1898 ± 0.13571	0.822
SII, mean ± sd	848.71 ± 769.26	845.76 ± 846.62	0.983
NLR, mean ± sd	3.6299 ± 3.2559	3.2943 ± 2.8278	0.541
PLR, mean ± sd	200.63 ± 97.205	197.92 ± 89.313	0.871
PNI, mean ± sd	44.205 ± 5.0166	45.463 ± 4.0214	0.130
LMR, mean ± sd	3.3942 ± 1.6958	3.5644 ± 1.6545	0.567
AGR, mean ± sd	1.4514 ± 0.28565	1.4695 ± 0.26745	0.714
Liver metastasis, n (%)			0.298
No	52 (59.8%)	35 (68.6%)	
Yes	35 (40.2%)	16 (31.4%)	
Peritoneal metastasis, n (%)			0.903
No	64 (73.6%)	38 (74.5%)	
Yes	23 (26.4%)	13 (25.5%)	
Tumor location, n (%)			0.870
L	22 (25.3%)	15 (29.4%)	
M	27 (31.0%)	15 (29.4%)	
U	38 (43.7%)	21 (41.2%)	
ECOG PS, n (%)			0.221
≤1	74 (85.1%)	47 (92.2%)	
>1	13 (14.9%)	4 (7.8%)	
Stages, n (%)			0.155
III	15 (17.2%)	14 (27.5%)	
IV	72 (82.8%)	37 (72.5%)	
Chemotherapy classification, n (%)			0.081
Platinum-based	71 (81.6%)	35 (68.6%)	
Non-platinum	16 (18.4%)	16 (31.4%)	

SII, systemic Immune-Inflammation Index; NLR, neutrophil to lymphocyte ratio; PLR, platelet to lymphocyte ratio; PNI, prognostic nutritional index; LMR, lymphocyte to monocyte Ratio; AGR, albumin to globulin ratio; ECOG PS, Eastern Cooperative Oncology Group Performance Status; SV, splenic volume.

### Increase in splenic volume after initiation of immunotherapy

The median baseline splenic volume for all enrolled patients was 166.1 cm³ (IQR: 133.80–212.94 cm³). The median final splenic volume after chemotherapy combined with immune checkpoint inhibitor treatment was 203.9 cm³ (IQR: 150.20–259.67 cm³). To assess whether splenic volume increased, a correlation analysis was performed between the two splenic volume measurements for the same subject. Paired sample boxplots showed a significant difference in splenic volume before and after immunotherapy in gastric cancer patients, with a notable increase (*P* < 0.001) (**Supplementary Figure 4**).

### Tumor response

The tumor response of the splenic volume increased group and the non-increased group is summarized in **Supplementary Table 3**. Overall, no significant differences were observed between the two groups in terms of short-term treatment outcomes. The ORR and DCR were 37.9% and 94.3% in the increased group, and 47.1% and 98.0% in the non-increased group, respectively, with no statistically significant differences (ORR, P = 0.293; DCR, P = 0.535). Notably, the proportion of patients with progressive disease (PD) was higher in the increased group than in the non-increased group (5.7% vs. 2.0%).

### Survival analysis

**Figure 2:**
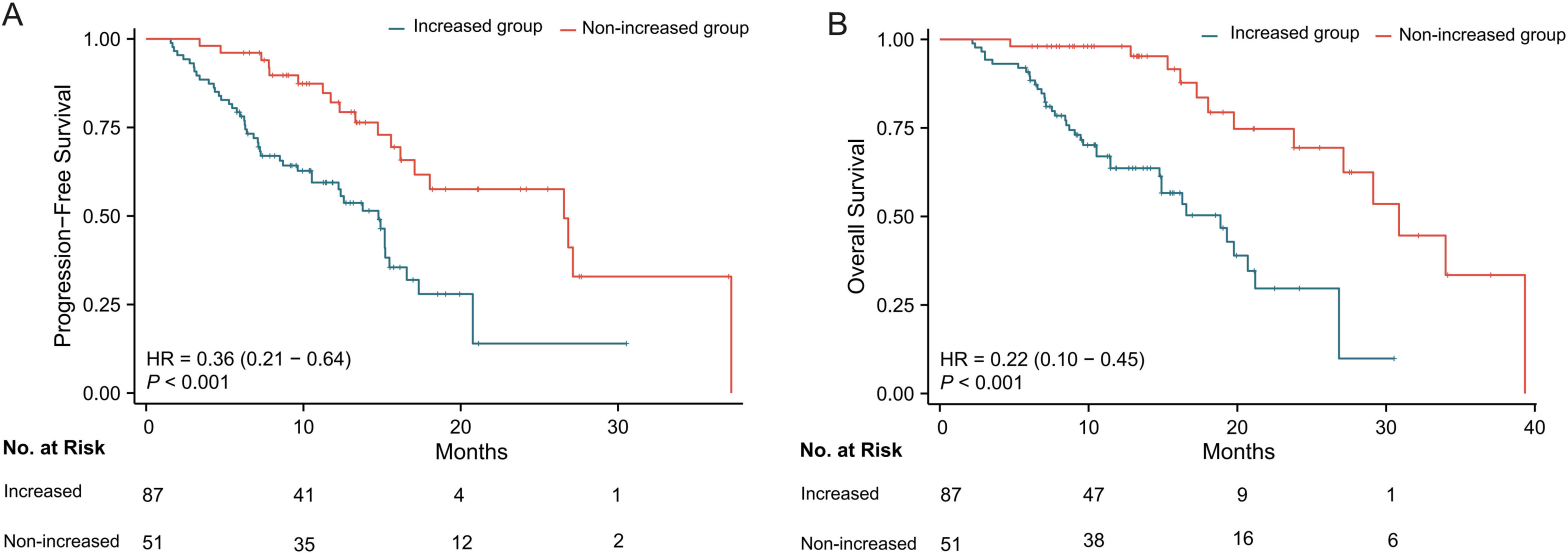
Kaplan-Meier survival analysis stratified by splenic volume change (ΔSV). **(A)** Progression-free survival (PFS). **(B)** Overall survival (OS). The increased ΔSV group (ΔSV >14%, red line) showed significantly shorter survival versus non-increased group (ΔSV ≤14%, blue line) (log-rank *P* < 0.001 for both).

Kaplan-Meier survival curves were plotted for PFS and OS in patients with ΔSV ≤ 14% and ΔSV > 14%. Log-rank tests showed that the “increased group” had significantly shorter PFS (14.8 months vs. 26.6 months, *P* < 0.001) and OS (18.9 months vs. 30.9 months, *P* < 0.001) compared to the “non-increased group” (**Figures 2A, B**). The HR for PFS in the non-increased group compared to the increased group was 0.36 (95% CI, 0.21–0.64), and the HR for OS was 0.22 (95% CI, 0.10–0.45). Additionally, time-dependent receiver operating characteristic (ROC) curves were used to explore the prognostic predictive value of splenic volume changes in advanced gastric cancer, showing that splenic volume change had good predictive value for both PFS and OS (**Supplementary Figure 5**). The area under the curve (AUC) for ΔSV was 0.711, 0.693, and 0.809 for 1-year, 1.5-year, and 2-year PFS, respectively, and 0.814, 0.729, and 0.808 for 1-year, 1.5-year, and 2-year OS, respectively.

### Univariate and multivariate analyses and subgroup analyses

Univariate Cox proportional hazards models were used to analyze demographic, clinical, and cancer-related factors (**Tables 2**, **3**). Specifically, in univariate regression analysis for PFS, Eastern Cooperative Oncology Group Performance Status (ECOG PS), PNI, splenic volume changes, and stages were associated with prognosis; for OS, ECOG PS, PNI, splenic volume changes, and stages were identified as potential predictors. These covariates were then included in multivariate regression analysis. In the multivariate analysis, higher ECOG PS (HR, 3.558 [95% CI, 1.801–7.029]; *P* < 0.001) and splenic volume increased (HR, 0.419 [95% CI, 0.233–0.751]; *P* = 0.004) were associated with a higher risk of disease progression. High PLR (HR, 2.143 [95% CI, 1.098–4.181]; *P* = 0.025), high ECOG scores (HR, 3.172 [95% CI, 1.509–6.669]; *P* = 0.002), stage IV disease (HR, 2.700 [95% CI, 1.067–6.829]; *P* = 0.025), and splenic volume increased (HR, 0.233 [95% CI, 0.109–0.501]; P< 0.001) were positively associated with shorter overall survival.

**Table 2:** Effect of altered splenic volume and prognostic factors on progression-free survival after immune checkpoint inhibitor therapy in univariate and multivariate Cox regression models. (n=138).

Characteristics	Total(N)	Univariate analysis		Multivariate analysis	
		Hazard ratio (95% CI)	P value	Hazard ratio (95% CI)	P value
Gender	138				
Male	95	Reference			
Female	43	0.738 (0.431 - 1.263)	0.267		
Age	138				
>60	68	Reference			
≤60	70	0.865 (0.530 - 1.410)	0.561		
Body mass index	138				
≤25	115	Reference			
>25	23	0.749 (0.380 - 1.475)	0.403		
Smoking	138				
No	116	Reference		Reference	
Yes	22	1.737 (0.939 - 3.215)	0.079	0.913 (0.463 - 1.802)	0.793
Hypertension	138				
No	108	Reference			
Yes	30	1.070 (0.581 - 1.969)	0.828		
SII	138				
≤438.36	40	Reference			
>438.36	98	1.029 (0.594 - 1.782)	0.919		
NLR	138				
≤2.88	79	Reference			
>2.88	59	1.126 (0.690 - 1.837)	0.636		
PLR	138				
≤153.75	51	Reference			
>153.75	87	1.334 (0.785 - 2.264)	0.286		
PNI	138				
>45.63	60	Reference		Reference	
≤45.63	78	1.902 (1.127 - 3.208)	**0.016**	1.425 (0.817 - 2.486)	0.212
LMR	138				
>3.61	50	Reference			
≤3.61	88	1.214 (0.721 - 2.042)	0.466		
ECOG PS	138				
≤1	121	Reference		Reference	
>1	17	4.766 (2.544 - 8.931)	**< 0.001**	3.558 (1.801 - 7.029)	**< 0.001**
Liver metastasis	138				
No	87	Reference			
Yes	51	1.177 (0.715 - 1.937)	0.521		
Peritoneal metastasis	138				
No	102	Reference			
Yes	36	0.970 (0.557 - 1.692)	0.916		
Tumor location	138				
L	37	Reference			
M	42	0.989 (0.510 - 1.915)	0.973		
U	59	1.039 (0.561 - 1.922)	0.904		
SV changes	138				
Increased	87	Reference		Reference	
Non-increased	51	0.362 (0.206 - 0.637)	**< 0.001**	0.419 (0.233 - 0.751)	**0.003**
Stages	138				
III	29	Reference		Reference	
IV	109	2.325 (1.146 - 4.720)	**0.019**	1.692 (0.808 - 3.544)	0.163
Chemotherapy classification	138				
Platinum-based	106	Reference			
Non-platinum	32	1.035 (0.559 - 1.914)	0.914		

SII, systemic Immune-Inflammation Index; NLR, neutrophil to lymphocyte ratio; PLR, platelet to lymphocyte ratio; PNI, prognostic nutritional index; LMR, lymphocyte to monocyte Ratio; AGR, albumin to globulin ratio; ECOG PS, Eastern Cooperative Oncology Group Performance Status; SV, splenic volume.

The bold values indicate statistically significant results (P < 0.05).

**Table 3:** Effect of altered splenic volume and prognostic factors on overall survival after immune checkpoint inhibitor therapy in univariate and multivariate Cox regression models. (n=138).

Characteristics	Total(N)	Univariate analysis		Multivariate analysis	
		Hazard ratio (95% CI)	P value	Hazard ratio (95% CI)	P value
Gender	138				
Male	95	Reference			
Female	43	0.729 (0.399 - 1.329)	0.302		
Age	138				
>60	68	Reference			
≤60	70	1.065 (0.615 - 1.843)	0.823		
Body mass index	138				
≤25	115	Reference			
>25	23	0.660 (0.307 - 1.419)	0.287		
Smoking	138				
No	116	Reference			
Yes	22	1.222 (0.545 - 2.743)	0.627		
Hypertension	138				
No	108	Reference			
Yes	30	1.104 (0.531 - 2.298)	0.791		
SII	138				
≤438.36	40	Reference			
>438.36	98	1.356 (0.706 - 2.605)	0.360		
NLR	138				
≤2.88	79	Reference			
>2.88	59	1.308 (0.753 - 2.273)	0.341		
PLR	138				
≤153.75	51	Reference		Reference	
>153.75	87	1.712 (0.922 - 3.180)	0.089	2.143 (1.098 - 4.181)	**0.025**
PNI	138				
>45.63	60	Reference		Reference	
≤45.63	78	2.215 (1.209 - 4.059)	**0.010**	1.457 (0.769 - 2.763)	0.248
LMR	138				
>3.61	50	Reference			
≤3.61	88	1.340 (0.743 - 2.419)	0.331		
ECOG PS	138				
≤1	121	Reference		Reference	
>1	17	4.546 (2.229 - 9.270)	**< 0.001**	3.172 (1.509 - 6.669)	**0.002**
Liver metastasis	138				
No	87	Reference			
Yes	51	0.992 (0.563 - 1.747)	0.978		
Peritoneal metastasis	138				
No	102	Reference			
Yes	36	1.180 (0.634 - 2.195)	0.602		
Tumor location	138				
L	37	Reference			
M	42	0.916 (0.452 - 1.857)	0.808		
U	59	0.734 (0.377 - 1.429)	0.362		
SV changes	138				
Increased	87	Reference		Reference	
Non-increased	51	0.216 (0.104 - 0.448)	**< 0.001**	0.233 (0.109 - 0.501)	**< 0.001**
Stages	138				
III	29	Reference		Reference	
IV	109	3.157 (1.331 - 7.491)	**0.009**	2.700 (1.067 - 6.829)	**0.036**
Chemotherapy classification	138				
Platinum-based	106	Reference			
Non-platinum	32	0.923 (0.447 - 1.907)	0.829		

SII, systemic Immune-Inflammation Index; NLR, neutrophil to lymphocyte ratio; PLR, platelet to lymphocyte ratio; PNI, prognostic nutritional index; LMR, lymphocyte to monocyte Ratio; AGR, albumin to globulin ratio; ECOG PS, Eastern Cooperative Oncology Group Performance Status; SV, splenic volume.

The bold values indicate statistically significant results (P < 0.05).

Internal validation of the multivariable Cox model incorporating ΔSV was performed. The apparent C-index of the model was 0.775. After bootstrap resampling, the optimism-corrected C-index was 0.756. Repeated 5-fold cross-validation yielded a mean C-index of 0.752 with a standard deviation of 0.020 (95% CI: 0.712–0.788). The internal validation results are summarized in **Supplementary Table 4**.

Sensitivity analyses were conducted to evaluate the effect of heterogeneous follow-up intervals on ΔSV estimation. Stratified analyses according to follow-up duration (<6 months, 6–12 months, and >12 months) yielded hazard ratios of comparable magnitude for both progression-free survival and overall survival. After correction using linear mixed-effects modeling, ΔSV remained significantly associated with survival outcomes. Similar results were observed after temporal standardization of splenic volume at a 6-month post-treatment time point. Detailed results are presented in **Supplementary Tables 5–8**.

Subgroup analyses based on baseline characteristics revealed consistent results for both PFS and OS. In both PFS and OS analyses (**Supplementary Figures 6, 7**), the risk of progression in the group with increased ΔSV was higher than that in the group with non-increased ΔSV, though some subgroups did not show statistical significance.

### Nonlinear relationship between splenic volume change and survival

**Figure 3:**
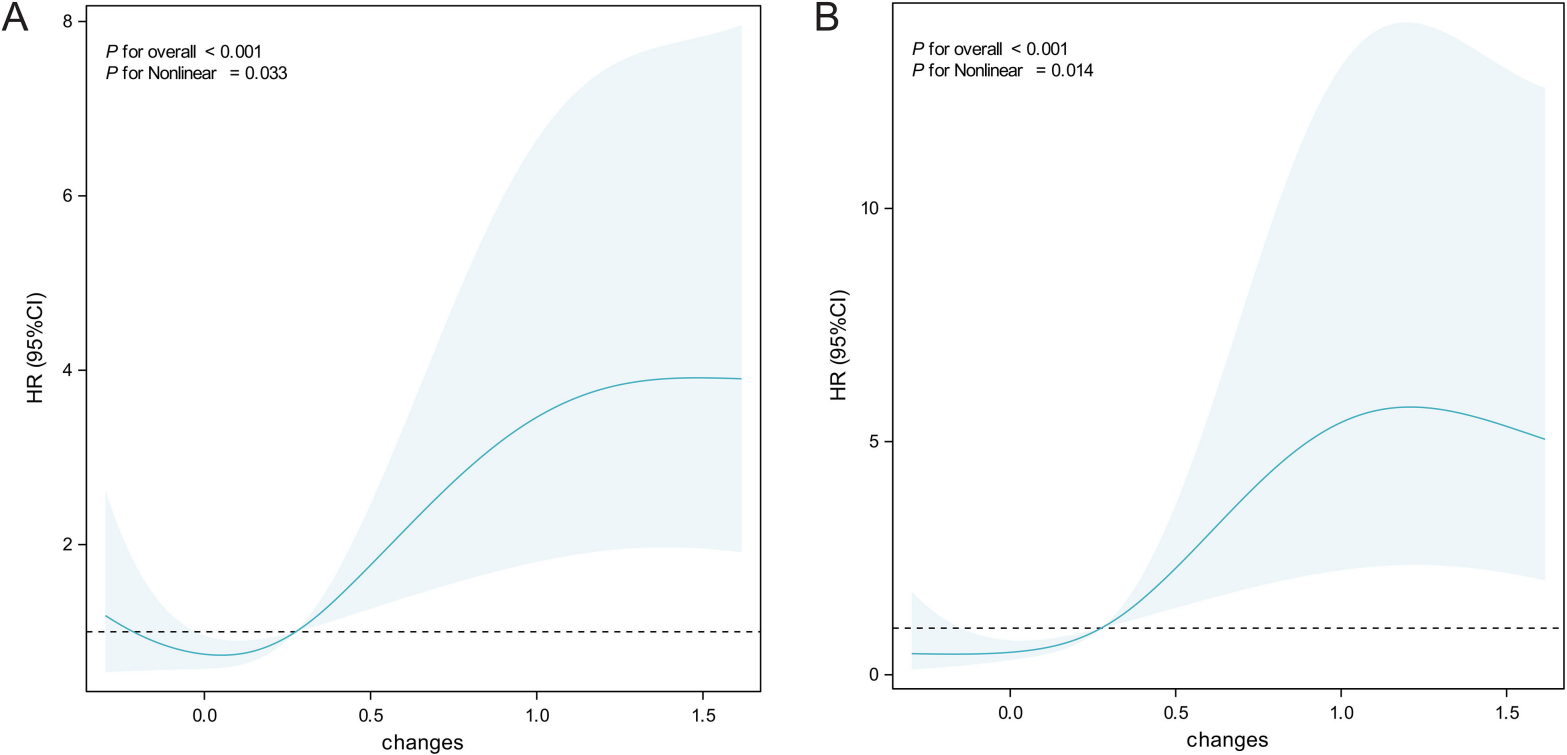
Restricted cubic spline analysis of ΔSV association with survival outcomes. **(A)** PFS. **(B)** OS. Hazard ratios (solid line) and 95% confidence intervals (shaded area) were adjusted for gender, age, BMI, hypertension, smoking, ECOG score, tumor location, and stage. Reference ΔSV=0% (dashed line).

After adjusting for covariates, we used restricted cubic spline (RCS) curves to explore the relationship between splenic volume change and PFS and OS. A significant nonlinear association was found between splenic volume change and PFS (**Figure 3A**) (overall *P* < 0.001, nonlinear *P* = 0.032). When the splenic volume change was small, the hazard ratio for disease progression remained low; as splenic volume increased, the risk of disease progression gradually rose. A similar nonlinear association was observed between splenic volume change and OS (**Figure 3B**) (overall *P* < 0.001, nonlinear *P* = 0.014). When splenic volume change was minimal, the hazard ratio for death was close to 1; as splenic volume increased, the hazard ratio rose rapidly, although the increase slowed over time, and the hazard ratio remained relatively high.

### Nomogram construction and calibration for prognosis prediction

**Figure 4:**
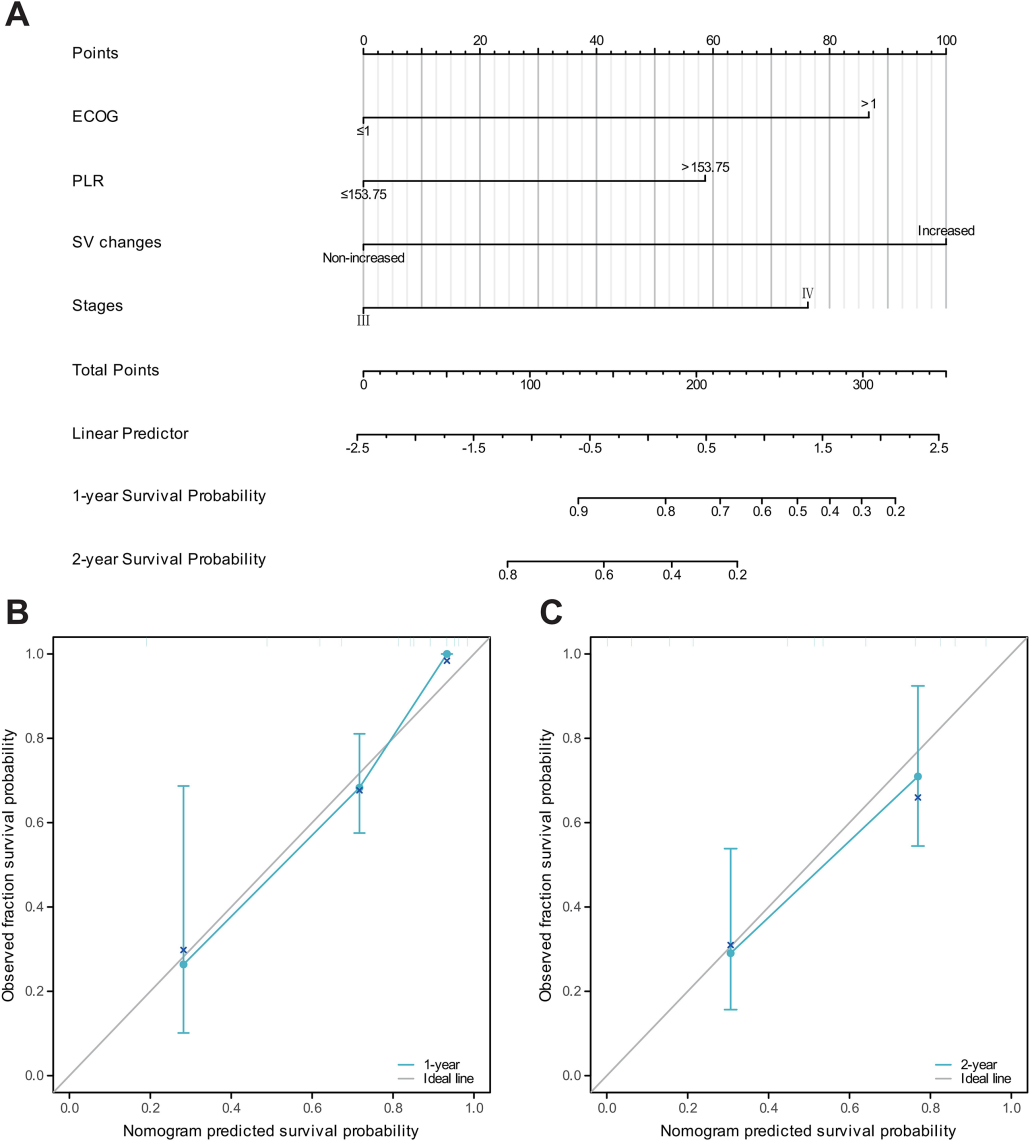
Nomogram development and validation for OS prediction. **(A)** Nomogram integrating ΔSV, stage, and clinical variables. Points are summed to predict 1-/2-year OS probability. **(B, C)** Calibration curves for **(B)** 1-year and **(C)** 2-year OS. X-axis: nomogram-predicted survival probability; Y-axis: actual Kaplan-Meier survival rate. Dotted line: ideal performance. Bars: 95% CI.

Based on the independent risk factors identified in the multivariate Cox regression, we combined ΔSV with key clinical features to create a nomogram for predicting 1-year and 2-year overall survival in patients with advanced gastric cancer (**Figure 4**). The predictive accuracy of the model was assessed using time-dependent AUC and calibration curves. The calibration curves confirmed a high consistency between predicted and actual outcomes. Compared with the time-dependent AUC curves for the single-factor stages prognosis, the multifactor prognostic model’s time-dependent AUC values for 1-year, 1.5-year, and 2-year survival were 0.802, 0.762, and 0.883, respectively, while the time-dependent AUC values for the single-factor stages model were only 0.564, 0.575, and 0.686 (**Supplementary Figure 8**).

Decision curve analysis was conducted to evaluate the net benefit of the nomogram for predicting 1-year and 2-year overall survival. As shown in **Supplementary Figure 9**, for 1-year overall survival, the nomogram yielded a higher net benefit than both the “treat-all” and “treat-none” strategies when the threshold probability was below approximately 0.65. For 2-year overall survival, the nomogram similarly outperformed the default strategies across threshold probabilities up to approximately 0.60.

## Discussion

To the best of our knowledge, this study is the first to report the association between ΔSV and both short-term treatment response and long-term prognosis in patients with GC receiving ICIs. Our primary finding demonstrates that patients with increased splenic volume after immunotherapy exhibited significantly shorter PFS and OS compared to those without splenic enlargement. Furthermore, multivariate analysis confirmed ΔSV as an independent prognostic risk factor, even after adjusting for ECOG PS and tumor stage. These results suggest that changes in spleen volume may reflect the dynamic remodeling of the immune microenvironment during immunotherapy and may serve as a novel radiological biomarker for prognostic evaluation in GC immunotherapy.

Previous studies have investigated the potential predictive value of baseline splenic volume and changes in splenic volume during treatment for various malignancies, including metastatic non-small cell lung cancer and metastatic renal cancer (14, 17). For instance, Aslan et al. reported that patients with metastatic renal cell carcinoma treated with ICIs showed a median splenic volume change of 10%, with greater changes in splenic volume correlating with poorer clinical outcomes, findings similar to those of the present study (14). In a study of metastatic colorectal cancer, Niogret et al. observed that a baseline splenic volume greater than 180 mL was strongly associated with poorer PFS. However, no statistically significant association was found for OS. Furthermore, an increase in splenic volume after 3 months of treatment did not significantly affect either PFS or OS. Notably, baseline splenic volume was significantly positively correlated with circulating levels of mononuclear cell MDSCs, highlighting the potential clinical significance of monitoring splenic volume during tumor treatment (18). In a related study of non-small cell lung cancer, Castagnoli et al. found no predictive or prognostic value of changes in splenic volume in pembrolizumab-treated patients (19). The discrepancies between our results and those of these studies may be attributed to differences in treatment methods and the types of diseases investigated.

The spleen, as a central immunological organ, plays a dual role in tumor immunity (20). On one hand, its red and white pulp structures provide a niche for T-cell activation and antigen presentation, potentially enhancing antitumor immunity (21, 22). On the other hand, excessive splenomegaly has been reported to be associated with the accumulation of immunosuppressive cell populations, such as MDSCs and Tregs, which may contribute to an immunosuppressive tumor microenvironment and reduced immunotherapeutic efficacy (23, 24). A potential mechanistic hypothesis is that changes in spleen volume may be associated with an imbalance between immune activation and immune exhaustion. Mild splenomegaly may reflect proliferation and activation of effector T cells (25), while excessive enlargement may indicate chronic inflammation or immune cell exhaustion, leading to diminished antitumor efficacy (26). Additionally, the spleen-bone marrow axis may play a role—spleen enlargement may stimulate the release of suppressive myeloid cells from the bone marrow, resulting in systemic immunosuppression (27–30).

Furthermore, MDSCs—heterogeneous populations of immunosuppressive myeloid cells—are central to tumor immune evasion, and their potential link with spleen volume changes offers a biologically plausible explanation for the observed clinical associations (31, 32). MDSCs inhibit T-cell activation through mechanisms involving arginase-1, reactive oxygen species (ROS), and immune checkpoint molecules such as PD-L1. They also promote Treg expansion, thereby systemically impairing antitumor immune responses (23). Under immunotherapeutic pressure, the spleen may serve as a reservoir for MDSCs (33, 34). However, in the absence of direct immune profiling, this interpretation remains speculative, and the observed associations should be regarded as correlative rather than mechanistically confirmed.

It should also be noted that all patients in this cohort received chemotherapy in combination with immune checkpoint inhibitors. Therefore, changes in splenic volume may reflect not only immune-mediated mechanisms but also chemotherapy-related effects, including hematopoietic suppression, inflammatory responses, or treatment-induced immune modulation. As such, ΔSV should be interpreted as an integrated imaging marker capturing systemic immune and inflammatory alterations during combination therapy rather than as a purely immune-specific indicator.

From a clinical perspective, ΔSV monitoring and the proposed nomogram are not intended to directly guide treatment continuation or modification. Instead, they are designed to support dynamic risk stratification and clinical surveillance during combination therapy. By integrating ΔSV with established clinical factors, the nomogram may help identify patients at higher risk of unfavorable outcomes, for whom closer follow-up or more frequent imaging assessment may be considered.

While our findings provide new insights into prognostic evaluation in gastric cancer immunotherapy, several limitations should be acknowledged. First, the retrospective nature of this study and its single-center design may introduce selection bias and limit the generalizability of our findings. Patients included in this cohort had available baseline and follow-up CT examinations, which may preferentially select individuals with more regular follow-up, potentially excluding those with incomplete assessments. Although we excluded patients with missing key data to ensure integrity, such exclusions may introduce bias. Additionally, heterogeneity in follow-up duration is inherent to retrospective cohorts and may influence the estimation and external applicability of ΔSV. Although sensitivity analyses were performed to assess the impact of this variability, residual bias cannot be fully excluded. Therefore, the prognostic value of ΔSV observed in this study warrants validation in larger, multicenter prospective studies.

Second, this study did not include peripheral blood or tissue-based immune profiling, which limited our ability to directly characterize immune cell populations within the spleen, such as myeloid-derived suppressor cells. As a result, mechanistic interpretations of the association between splenic volume changes and clinical outcomes remain correlative rather than causative. Future studies should incorporate multicenter prospective cohorts and integrate advanced immunological profiling techniques, including single-cell sequencing and cytokine analyses, to validate the predictive utility of ΔSV, elucidate its underlying biological mechanisms, and explore potential therapeutic strategies targeting the splenic immune microenvironment.

## Conclusions

In gastric cancer patients receiving chemotherapy combined with ICIs, splenic volume tends to increase, and this increase is often associated with poorer PFS and OS. CT-based monitoring of splenic volume may provide additional prognostic information during combination therapy and warrants further validation in prospective studies.

## Data Availability

The raw data supporting the conclusions of this article will be made available by the authors, without undue reservation.
