# Supplementary material for: Association between splenic volume changes and prognosis in advanced gastric cancer patients receiving chemotherapy combined with immunotherapy
Source: Front Oncol. 2026 Feb 11;16:1763993. doi: 10.3389/fonc.2026.1763993 (PMC12932240; doi:10.3389/fonc.2026.1763993)
Supplement: Supplementary file 1 [file DataSheet1.docx]

**SUPPLEMENTAL MATERIAL**

**Supplementary Tables**

**Supplementary Table 1.** Optimal cut-off values of baseline blood parameters determined using the maximally selected log-rank statistic.

**Supplementary Table 2.** Baseline characteristics of the study population stratified by baseline splenic volume.

**Supplementary Table 3.** Tumor response comparison between the SV-increased and SV-non-increased groups.

**Supplementary Table 4.** Internal validation of the multivariable Cox model incorporating ΔSV.

**Supplementary Table 5.** Sensitivity analyses assessing the robustness of the association between ΔSV and progression-free survival under heterogeneous follow-up durations.

**Supplementary Table 6.** Multivariable Cox regression analysis for progression-free and overall survival using LMM-corrected ΔSV.

**Supplementary Table 7.** Multivariable Cox regression analysis for progression-free and overall survival after 6-month temporal standardization of ΔSV.

**Supplementary Table 8.** Statistical characteristics of linear mixed-effects modeling and temporal standardization for ΔSV estimation.

**Supplementary Figures**

**Supplementary Figure 1.** Determination of the optimal cut-off point for changes in splenic volume using the maximally selected log-rank statistic.

**Supplementary Figure 2.** Bland–Altman plot for inter-observer agreement of splenic volume measurements.

**Supplementary Figure 3.** Kaplan–Meier survival curves for progression-free survival and overall survival stratified by median baseline splenic volume.

**Supplementary Figure 4.** Box plot of changes in splenic volume during immunotherapy.

**Supplementary Figure 5.** Time-dependent ROC curves for progression-free and overall survival at 1, 1.5, and 2 years.

**Supplementary Figure 6.** Forest plot of subgroup analysis for progression-free survival according to ΔSV status.

**Supplementary Figure 7.** Forest plot of subgroup analysis for overall survival according to ΔSV status.

**Supplementary Figure 8.** Comparison of time-dependent AUC curves between the multifactor prognostic model (ECOG, PLR, ΔSV, and stage) and the stage-only model.

**Supplementary Figure 9.** Decision curve analysis showing the net benefit of the nomogram for predicting 1-year and 2-year overall survival compared with treat-all and treat-none strategies.

**Supplementary Table 1.** **The optimal cut - off value of baseline blood parameters selected based on the maximally selected log - rank statistic.**

| Characteristics | SII | NLR | PLR | PNI | LMR | SV | △SV |
| --- | --- | --- | --- | --- | --- | --- | --- |
| cut-off value | 438.36 | 2.88 | 153.75 | 45.63 | 3.61 | 1.52 | 160.03 |

SII, systemic Immune-Inflammation Index; NLR, neutrophil to lymphocyte ratio; PLR, platelet to lymphocyte ratio; PNI, prognostic nutritional index; LMR, lymphocyte to monocyte Ratio; SV, splenic volume.

**Supplementary Table 2. Baseline characteristics of the study population stratified according to baseline splenic volume. (n=138)**

| Characteristics | SV≤160.03 | SV>160.03 | P value |
| --- | --- | --- | --- |
| n | 61 | 77 |  |
| Gender, n (%) |  |  | 0.268 |
| Male | 39 (63.9%) | 56 (72.7%) |  |
| Female | 22 (36.1%) | 21 (27.3%) |  |
| Age, n (%) |  |  | 0.090 |
| >60 | 35 (57.4%) | 33 (42.9%) |  |
| ≤60 | 26 (42.6%) | 44 (57.1%) |  |
| Body mass index, n (%) |  |  | 0.702 |
| ≤25 | 50 (82%) | 65 (84.4%) |  |
| >25 | 11 (18%) | 12 (15.6%) |  |
| Smoking, n (%) |  |  | 0.045 |
| No | 47 (77%) | 69 (89.6%) |  |
| Yes | 14 (23%) | 8 (10.4%) |  |
| Hypertension, n (%) |  |  | 0.255 |
| No | 45 (73.8%) | 63 (81.8%) |  |
| Yes | 16 (26.2%) | 14 (18.2%) |  |
| Cr, mean ± sd | 70.833 ± 20.466 | 77.016 ± 72.081 | 0.517 |
| Blood urea nitrogen, mean ± sd | 5.1018 ± 1.5971 | 5.3721 ± 2.5666 | 0.473 |
| Alkaline phosphatase, mean ± sd | 145.82 ± 209.16 | 106.29 ± 80.983 | 0.167 |
| Ca, mean ± sd | 2.1993 ± 0.14496 | 2.176 ± 0.14187 | 0.345 |
| SII, mean ± sd | 684.12 ± 491.79 | 977.14 ± 954.87 | 0.021 |
| NLR, mean ± sd | 2.8525 ± 1.5358 | 4.0235 ± 3.8507 | 0.017 |
| PLR, mean ± sd | 189.89 ± 90.621 | 207.34 ± 96.558 | 0.281 |
| PNI, mean ± sd | 45.188 ± 4.9204 | 44.26 ± 4.5041 | 0.251 |
| LMR, mean ± sd | 3.5961 ± 1.8558 | 3.347 ± 1.5234 | 0.388 |
| AGR, mean ± sd | 1.4639 ± 0.25307 | 1.4536 ± 0.2982 | 0.830 |
| Radiation therapy, n (%) |  |  | 0.728 |
| No | 46 (75.4%) | 60 (77.9%) |  |
| Yes | 15 (24.6%) | 17 (22.1%) |  |
| Liver metastasis, n (%) |  |  | 0.208 |
| No | 42 (68.9%) | 45 (58.4%) |  |
| Yes | 19 (31.1%) | 32 (41.6%) |  |
| Peritoneal metastasis, n (%) |  |  | 0.671 |
| No | 44 (72.1%) | 58 (75.3%) |  |
| Yes | 17 (27.9%) | 19 (24.7%) |  |
| Tumor location, n (%) |  |  | 0.931 |
| L | 17 (27.9%) | 20 (26%) |  |
| M | 19 (31.1%) | 23 (29.9%) |  |
| U | 25 (41%) | 34 (44.2%) |  |
| ECOG PS, n (%) |  |  | 0.788 |
| ≤1 | 54 (88.5%) | 67 (87%) |  |
| >1 | 7 (11.5%) | 10 (13%) |  |
| Stages, n (%) |  |  | 0.181 |
| Ⅲ | 16 (26.2%) | 13 (16.9%) |  |
| Ⅳ | 45 (73.8%) | 64 (83.1%) |  |

SII, systemic Immune-Inflammation Index; NLR, neutrophil to lymphocyte ratio; PLR, platelet to lymphocyte ratio; PNI, prognostic nutritional index; LMR, lymphocyte to monocyte Ratio; AGR, albumin to globulin ratio；ECOG PS, Eastern Cooperative Oncology Group Performance Status.

**Supplementary Table 3. Tumor Response Between the SV-Increased Group and SV-Non-increased Group.**

| Characteristics | Increased group | Non-increased group | P value |
| --- | --- | --- | --- |
| CR | 0 | 0 |  |
| PR | 33 (37.9%) | 24 (47.1%) |  |
| SD | 49 (56.3%) | 26 (51.0%) |  |
| PD | 5 (5.7%) | 1 (2.0%) |  |
| ORR | 33 (37.9%) | 24 (47.1%) | 0.293 |
| DCR | 82 (94.3%) | 50 (98.0%) | 0.535 |

CR, complete response; PR, partial response; SD, stable disease; PD, progressive disease; ORR, objective response rate; DCR, disease control rate.

# Supplementary Table 4. Internal validation of the multivariable Cox model incorporating ΔSV

| Validation method | C-index | Additional information |
| --- | --- | --- |
| Apparent performance (training set) | 0.775 | Dxy = 0.5507 |
| Bootstrap-corrected (B = 1000) | 0.756 | Optimism-corrected Dxy = 0.5118 |
| Repeated 5-fold cross-validation (R = 50) | 0.752 ± 0.020 | 95% CI: 0.712–0.788 |

CV, cross-validation; CI, confidence interval; Dxy, Somers’ D rank correlation.
Internal validation was performed to assess the robustness and potential overfitting of the multivariable Cox model incorporating ΔSV. Bootstrap resampling was conducted with 1,000 repetitions to estimate optimism-corrected model performance. Discrimination stability was further evaluated using repeated 5-fold cross-validation (50 repetitions).

**Supplementary Table 5. Sensitivity analyses assessing the robustness of ΔSV–PFS association under heterogeneous follow-up durationsity Analyses for Follow-up Duration Variability (PFS)**

| Analysis Type | Sample Size (n) | Events (n) | Hazard Ratio (HR) | P-value |
| --- | --- | --- | --- | --- |
| Main analysis (full cohort) | 138 | 66 | 2.46 | 0.002 |
| Stratified: <6 months | 45 | 26 | 2.4 | 0.16 |
| Stratified: 6–12 months | 44 | 15 | 2.92 | 0.078 |
| Stratified: >12 months | 49 | 25 | 2.4 | 0.049 |
| LMM-corrected ΔSV | 138 | 66 | 2.46 | 0.002 |
| 6-month interpolation–corrected ΔSV | 93 | 40 | 2.13 | 0.034 |

PFS, progression-free survival; HR, hazard ratio; LMM, linear mixed-effects model.

Hazard ratios were estimated using Cox proportional hazards regression. Stratified analyses were performed according to CT follow-up duration (<6 months, 6–12 months, and >12 months). LMM-corrected ΔSV values were derived using a linear mixed-effects model accounting for individual follow-up variability. Temporal standardization was performed by interpolating splenic volume at a uniform 6-month post-treatment time point.

**Supplementary Table 6A. Multivariable Cox regression analysis for PFS using LMM-corrected ΔSV (n = 138)**

| Variable | Coefficient | HR | 95% CI | P-value |
| --- | --- | --- | --- | --- |
| ΔSV (LMM-corrected, binary) | 0.9 | 2.46 | 1.39–4.35 | 0.002 |
| ECOG PS ≤1 | -1.298 | 0.27 | 0.14–0.52 | <0.001 |
| Stage IV | 0.617 | 1.85 | 0.89–3.84 | 0.097 |

PFS, progression-free survival; LMM, linear mixed-effects model; HR, hazard ratio; CI, confidence interval; ECOG PS, Eastern Cooperative Oncology Group performance status.

ΔSV values were corrected for follow-up duration using a linear mixed-effects model with random intercepts and slopes. Variables shown were included in the multivariable Cox regression model. Model discrimination was assessed using Harrell’s concordance index (C-index).

**Supplementary Table 6B. Multivariable Cox regression analysis for OS using LMM-corrected ΔSV (n = 138)**

| Variable | Coefficient | HR | 95% CI | P-value |
| --- | --- | --- | --- | --- |
| ΔSV (LMM-corrected, binary) | 1.389 | 4.01 | 1.91–8.44 | <0.001 |
| ECOG PS ≤1 | -1.134 | 0.32 | 0.16–0.66 | 0.002 |
| Stage IV | 0.901 | 2.46 | 1.01–6.02 | 0.048 |

OS, overall survival; LMM, linear mixed-effects model; HR, hazard ratio; CI, confidence interval; ECOG PS, Eastern Cooperative Oncology Group performance status.

ΔSV values were corrected for follow-up duration using a linear mixed-effects model. Variables shown were included in the multivariable Cox regression model. Model discrimination was assessed using Harrell’s concordance index (C-index).

**Supplementary Table 7A. Multivariable Cox regression analysis for PFS after 6-month temporal standardization of ΔSV (n = 93)**

| Variable | Coefficient | HR | 95% CI | P-value |
| --- | --- | --- | --- | --- |
| ΔSV (6-month interpolated, binary) | 0.755 | 2.13 | 1.06–4.28 | 0.034 |
| ECOG PS ≤1 | -0.828 | 0.44 | 0.14–1.34 | 0.146 |
| Stage IV | 0.639 | 1.89 | 0.82–4.36 | 0.133 |

PFS, progression-free survival; HR, hazard ratio; CI, confidence interval; ECOG PS, Eastern Cooperative Oncology Group performance status.

Temporal standardization was performed by interpolating splenic volume at a uniform 6-month post-treatment time point. Patients with follow-up shorter than 6 months were excluded to avoid extrapolation bias. Variables shown were included in the multivariable Cox regression model.

**Supplementary Table 7B. Multivariable Cox regression analysis for OS after 6-month temporal standardization of ΔSV (n = 93)**

| Variable | Coefficient | HR | 95% CI | P-value |
| --- | --- | --- | --- | --- |
| ΔSV (6-month interpolated, binary) | 1.137 | 3.12 | 1.36–7.16 | 0.007 |
| ECOG PS ≤1 | -0.257 | 0.77 | 0.17–3.51 | 0.74 |
| Stage IV | 1.108 | 3.03 | 1.03–8.91 | 0.044 |

OS, overall survival; HR, hazard ratio; CI, confidence interval; ECOG PS, Eastern Cooperative Oncology Group performance status.

Temporal standardization was performed by interpolating splenic volume at a uniform 6-month post-treatment time point. Patients with insufficient follow-up duration were excluded to avoid extrapolation bias. Variables shown were included in the multivariable Cox regression model.

**Supplementary Table 8. Statistical characteristics of linear mixed-effects modeling and temporal standardization for ΔSV estimation**

| Metric | Value |
| --- | --- |
| Number of patients | 138 |
| Total observations | 276 |
| ICC for repeated spleen volume measurements | 0.724 |
| Fixed effect (per 1-month follow-up) | +1.95 cm³ (P < 0.001) |
| Random effects variance | 4749 |
| Residual variance | 1807 |

LMM, linear mixed-effects model; ICC, intraclass correlation coefficient.

The linear mixed-effects model was fitted using repeated splenic volume measurements to account for follow-up duration variability. ICC reflects the reproducibility of repeated splenic volume measurements. Temporal standardization was performed at a 6-month post-treatment time point, and correlation analysis was used to assess consistency between original and interpolated ΔSV values.


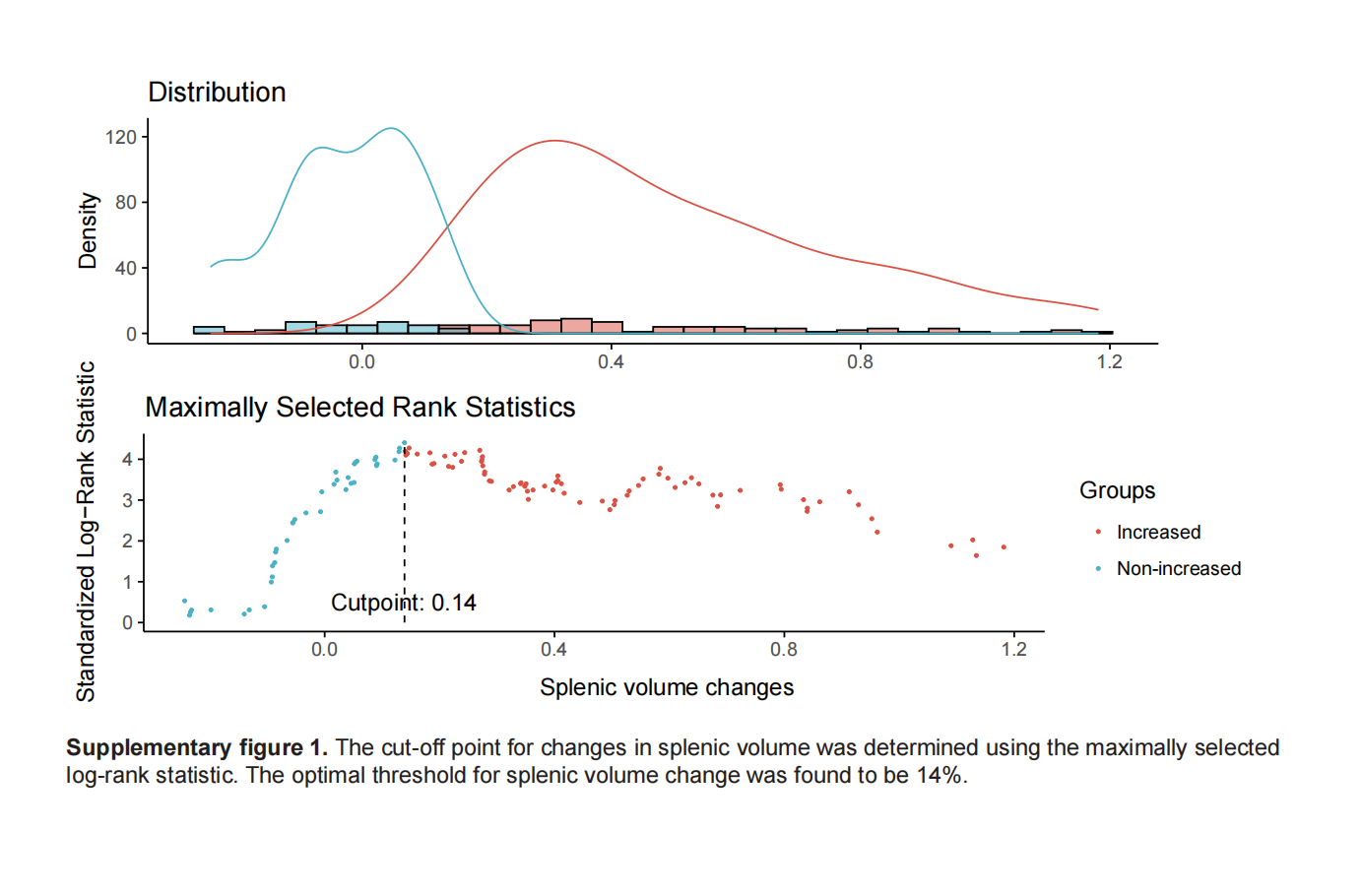


**Supplementary figure 1.** The cut-off point for changes in splenic volume was determined using the maximally selectedlog-rank statistic. The optimal threshold for splenic volume change was found to be 14%.


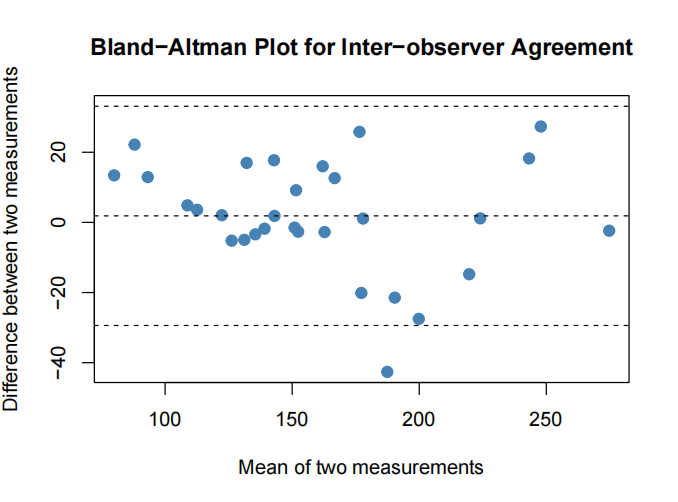


**Supplementary figure 2.** Bland-Altman plot for inter-observer agreement of splenic volume measurements.

Bland-Altman plot showing inter-observer agreement of CT-derived splenic volume measurements in 30 randomly selected patients. Differences between measurements from two independent radiologists are plotted against their mean values. The solid line indicates the mean difference, and the dashed lines represent the 95% limits of agreement.


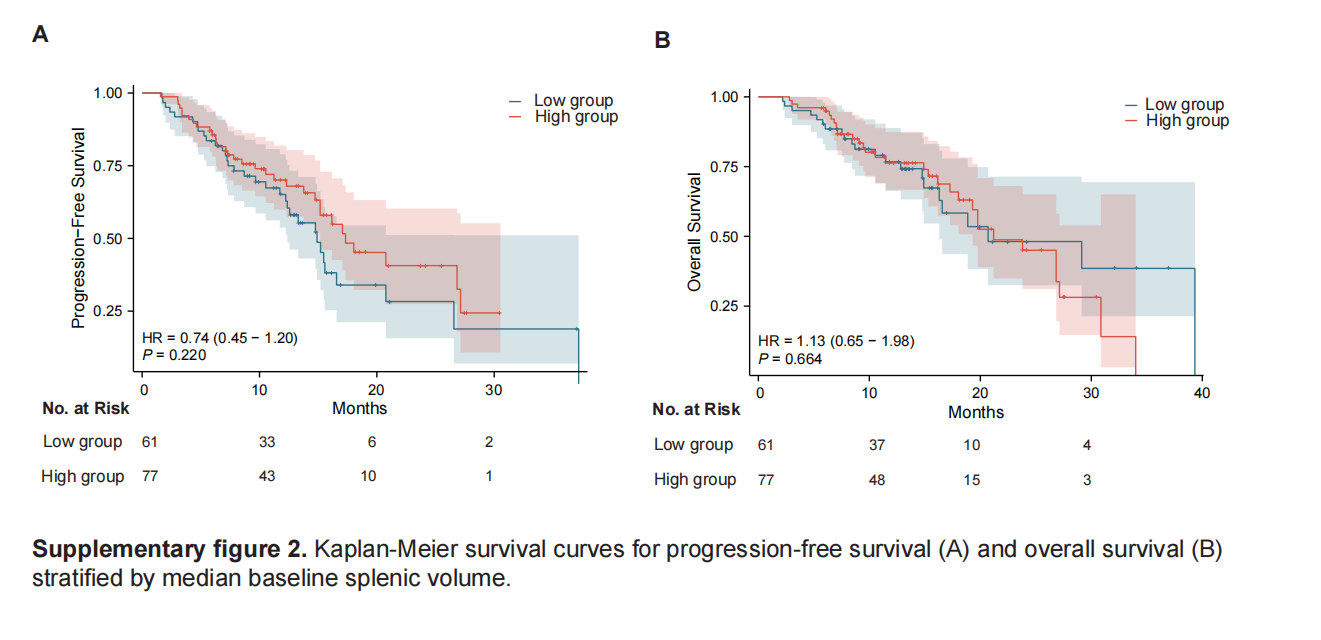


**Supplementary figure 3.** Kaplan-Meier survival curves for progression-free survival (A) and overall survival (B)stratified by median baseline splenic volume.


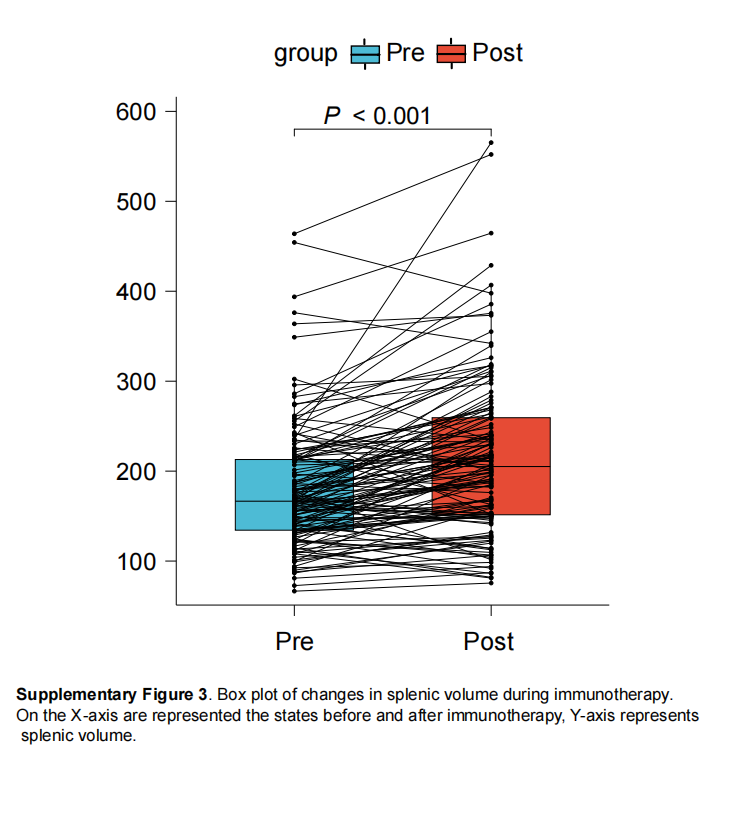


**Supplementary Figure 4.** Box plot of changes in splenic volume during immunotherapy.On the X-axis are represented the states before and after immunotherapy, Y-axis representssplenic volume.


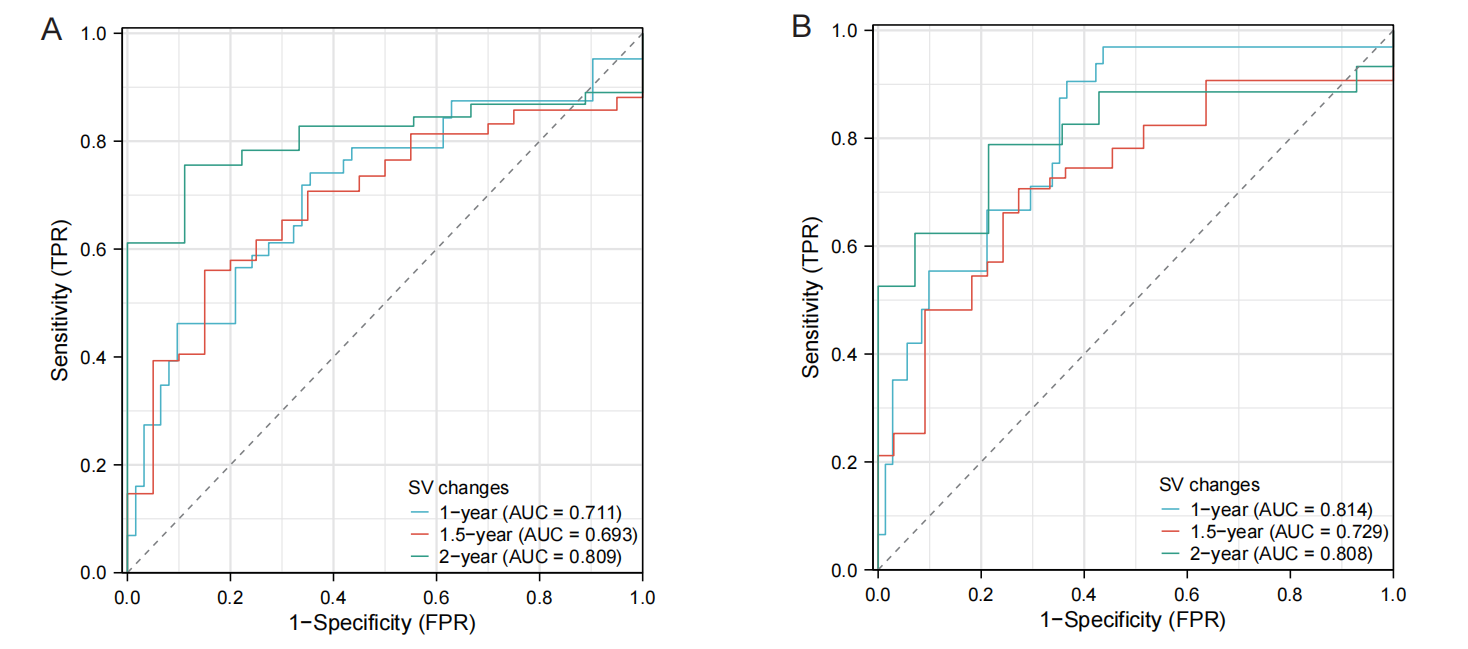


**Supplementary Figure 5.** Time-dependant ROC curves for PFS（A） and OS（B） at 1 year, 1.5 years and 2 years. ROC, receiver operating characteristic; PFS, progression-free survival; OS, overall survival; AUC, area under the curve; TPR, true positive rate; FPR, false positive rate.


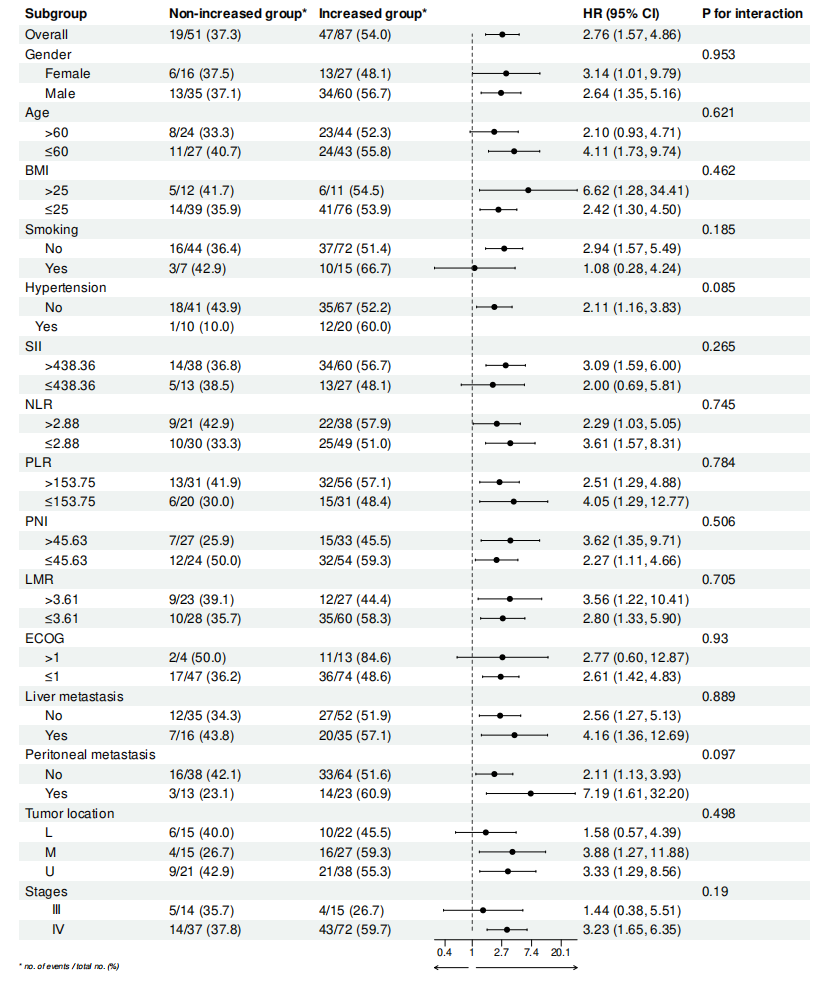
**Supplementary Figure 6.** Forest plot of subgroup analysis in progression-free survival between the △SV-increased group and △SV-non-increased group. Dashed line indicates Hazard ratio of 1. HR, hazard ratio; CI, confidence interval; SII, systemic Immune-Inflammation Index; NLR, neutrophil to lymphocyte ratio; PLR, platelet to lymphocyte ratio; PNI, prognostic nutritional index; LMR, lymphocyte to monocyte Ratio; AGR, albumin to globulin ratio; ECOG PS, Eastern Cooperative Oncology Group Performance Status; SV, splenic volume.


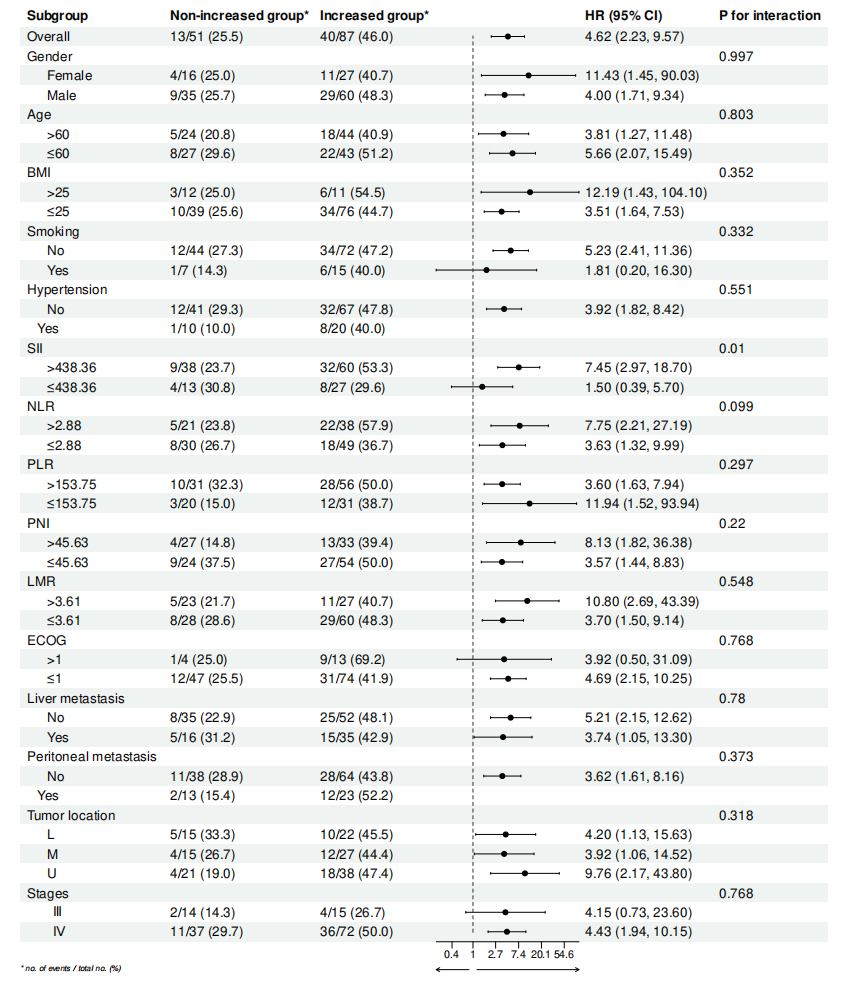
**Supplementary Figure 7.** Forest plot of subgroup analysis in overall survival between the △SV-increased group and △SV-non-increased group. Dashed line indicates Hazard ratio of 1. HR, hazard ratio; CI, confidence interval; SII, systemic Immune-Inflammation Index; NLR, neutrophil to lymphocyte ratio; PLR, platelet to lymphocyte ratio; PNI, prognostic nutritional index; LMR, lymphocyte to monocyte Ratio; AGR, albumin to globulin ratio; ECOG PS, Eastern Cooperative Oncology Group Performance Status; SV, splenic volume.


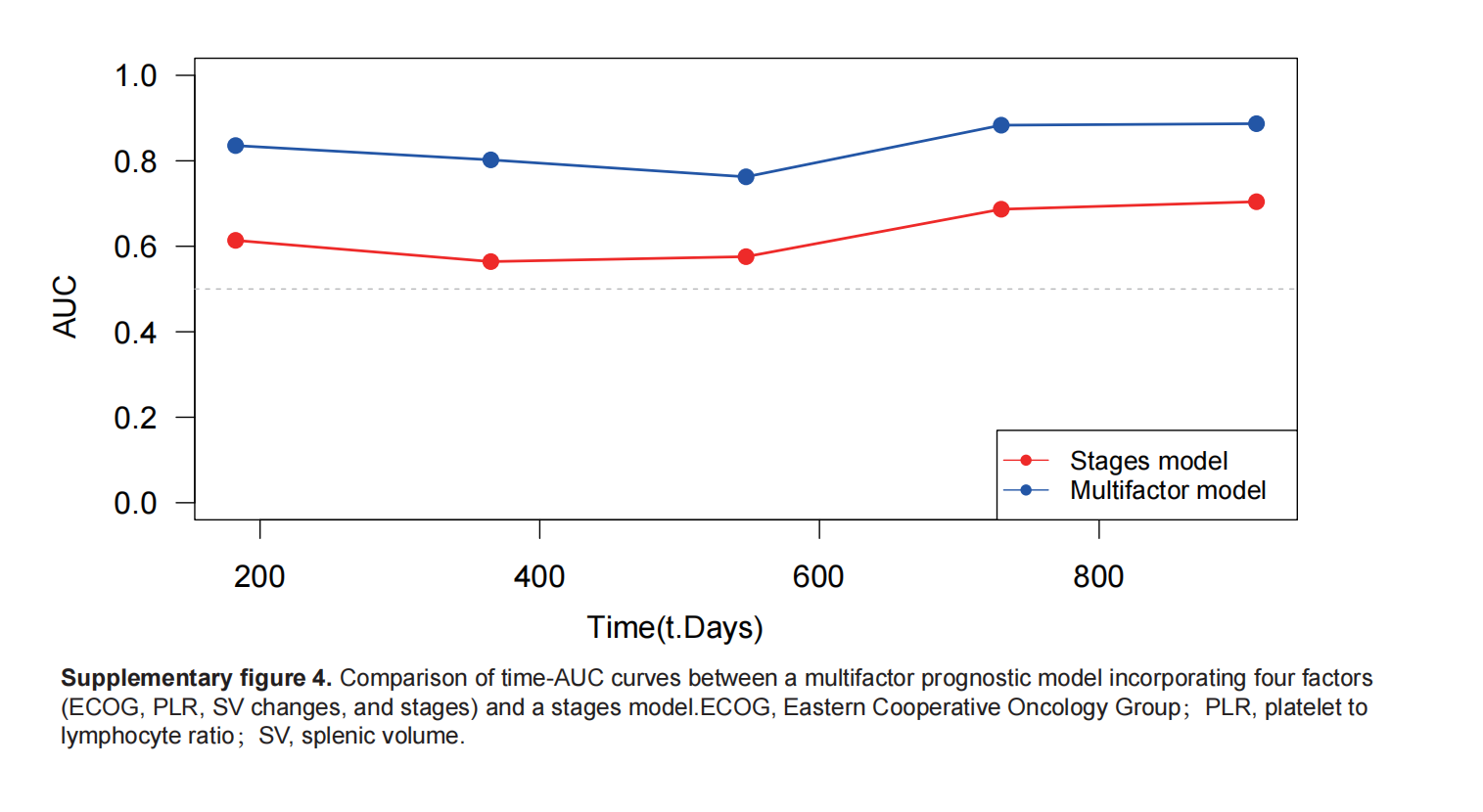


**Supplementary figure 8.** Compaison of time-AUC curves between a multifactor prognostic model incorporating four factors(ECOG, PLR, SV changes, and stages) and a stages model.ECOG, Eastem Cooperative Oncology Group; PLR, platelet tolymphocyte ratio: Sv, splenic volume


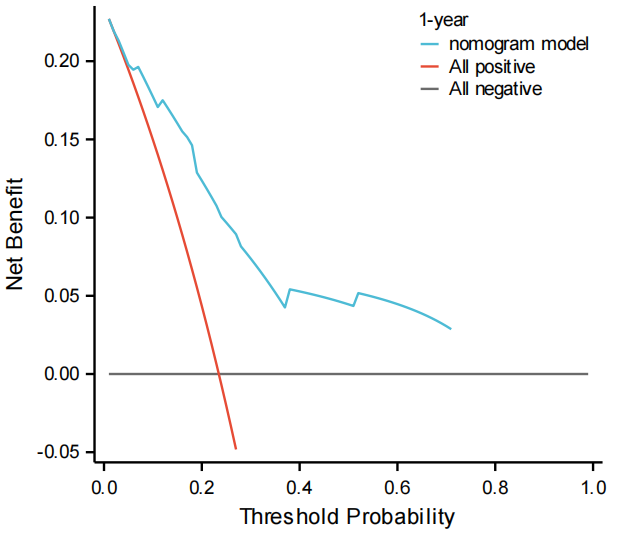

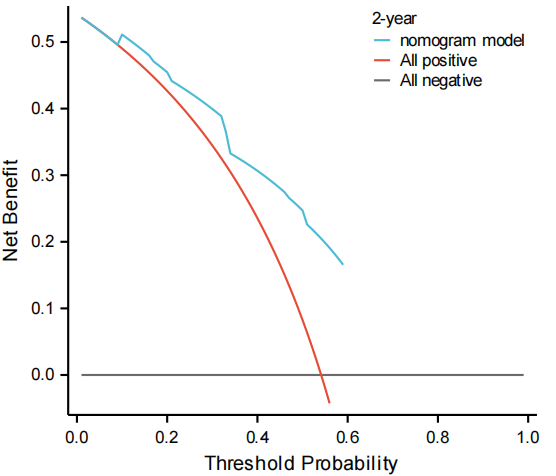


**Supplementary figure 9.** Decision curve analysis showing the net benefit of the nomogram for 1-year and 2-year overall survival compared with treat-all and treat-none strategies.
